# Nuclear Magnetic Resonance Metabolomic Profiling of Mouse Kidney, Urine and Serum Following Renal Ischemia/Reperfusion Injury

**DOI:** 10.1371/journal.pone.0163021

**Published:** 2016-09-22

**Authors:** François Jouret, Justine Leenders, Laurence Poma, Jean-Olivier Defraigne, Jean-Marie Krzesinski, Pascal de Tullio

**Affiliations:** 1 Division of Nephrology, University of Liège Hospital (ULg CHU), Liège, Belgium; 2 Groupe Interdisciplinaire de Génoprotéomique Appliquée (GIGA), Cardiovascular Sciences, University of Liège, Liège, Belgium; 3 Centre for Interdisciplinary Research on Medicines (CIRM), University of Liège, Liège, Belgium; Instituto de Investigacion Sanitaria INCLIVA, SPAIN

## Abstract

**Background:**

Ischemia/reperfusion (I/R) is the most common cause of acute kidney injury (AKI). Its pathophysiology remains unclear. Metabolomics is dedicated to identify metabolites involved in (patho)physiological changes of integrated living systems. Here, we performed ^1^H-Nuclear Magnetic Resonance metabolomics using urine, serum and kidney samples from a mouse model of renal I/R.

**Methods:**

Renal 30-min ischemia was induced in 12-week-old C57BL/6J male mice by bilaterally clamping vascular pedicles, and was followed by 6, 24 or 48-hour reperfusion (n = 12/group). Sham-operated mice were used as controls. Statistical discriminant analyses, i.e. principal component analysis and orthogonal projections to latent structures (OPLS-DA), were performed on urine, serum and kidney lysates at each time-point. Multivariate receiver operating characteristic (ROC) curves were drawn, and sensitivity and specificity were calculated from ROC confusion matrix (with averaged class probabilities across 100 cross-validations).

**Results:**

Urine OPLS-DA analysis showed a net separation between I/R and sham groups, with significant variations in levels of taurine, di- and tri-methylamine, creatine and lactate. Such changes were observed as early as 6 hours *post* reperfusion. Major metabolome modifications occurred at 24h *post* reperfusion. At this time-point, correlation coefficients between urine spectra and conventional AKI biomarkers, i.e. serum creatinine and urea levels, reached 0.94 and 0.95, respectively. The area under ROC curve at 6h, 24h and 48h *post* surgery were 0.73, 0.98 and 0.97, respectively. Similar discriminations were found in kidney samples, with changes in levels of lactate, fatty acids, choline and taurine. By contrast, serum OPLS-DA analysis could not discriminate sham-operated from I/R-exposed animals.

**Conclusions:**

Our study demonstrates that renal I/R in mouse causes early and sustained metabolomic changes in urine and kidney composition. The most implicated pathways at 6h and 24h *post* reperfusion include gluconeogenesis, taurine and hypotaurine metabolism, whereas protein biosynthesis, glycolysis, and galactose and arginine metabolism are key at 48h *post* reperfusion.

## Introduction

Ischemia/reperfusion (I/R) is the primary cause of acute kidney injury (AKI), a common situation currently defined as a rapid fall of glomerular filtration rate (GFR) and/or a decline in urine output [1]. The incidence of AKI is generally 5–7.5% in all acute care hospitalizations, but it accounts for up to 20% of admissions to intensive care units [2]. Furthermore, approximately 30–40% of all cases of AKI during hospitalization are observed in operative settings, and particularly after cardiovascular surgery [3]. The reduction or interruption of renal perfusion with a subsequent reflow induces significant cell metabolism perturbations and tissue inflammation [4–6]. Still, our understanding of the pathophysiology of I/R-associated AKI remains limited, which most often delays the diagnostic procedure and limits the therapeutic options.

The metabolomic approach consists in holistically characterizing metabolite abundances and/or fluctuations in biological matrices [7, 8]. According to the Metabolomics Society (http://www.metabolomicssociety.org/), the term “metabolomics” corresponds to “the comprehensive characterization of the small molecule metabolites in biological systems which can provide an overview of the metabolic status and global biochemical events associated with a cellular or biological system” [9, 10]. Metabolomics has been applied to mammalian systems biology to study the health-disease continuum, the homeostatic impact of certain diets and the safety/efficacy profile of drug therapy. Furthermore, such a global profiling of metabolites appears particularly useful to identify novel prognosis and diagnosis biomarkers, and innovative targets for drug discovery [11]. Metabolomics provides unique, challenging opportunities to link dynamic variations of the metabolome with a physiological or a pathological status and offers an innovative global insight into the relationships between genes, gene expression, environment, phenotype, lifestyle and pathologies. Moreover, the comprehension of the mechanisms that underlie the transition from physiological to pathophysiological states is of great interest for the discovery and the development of therapeutic strategies [12].

Technological developments are one of the main driving forces in scientific knowledge. Recent advances in two analytical platforms of mass spectrometry (MS) and Nuclear Magnetic Resonance (NMR) spectroscopy have put forward the discipline of metabolomics. Each of these techniques has advantages and limitations (for a detailed review see Ref.[13]). Despite a lower sensitivity in comparison to MS, NMR spectroscopy benefits from being non-destructive, quantitative, highly reproducible and fast (acquisition of metabolite profiles within 10 min per sample), with minimal sample preparation. This technics is particularly adapted to analyze biofluid collections, like urine or blood, but also tissue after a lysis step. Therefore, even if NMR spectroscopy can only detect and reliably quantify metabolites present in high concentrations [13, 14], this approach leads to a non-targeted snapshot of the metabolic pattern linked to a physiologic or a pathologic state.

In nephrology, metabolomics was first used to detect metabolome changes associated with drug-induced AKI [15, 16]. Interestingly, recent reports based on murine and human models of renal I/R highlighted relevant changes in blood and kidney metabolomes at the time of both injury and recovery. Most of these studies used MS-based metabolomic approach and led to the structural documentation of several endogenous metabolites whose abundance were modified by renal I/R [17–19]. ^1^H-NMR metabolomics has been used to evaluate drug-induced nephrotoxicity or the impact of cold ischemia at the time of kidney transplantation [20–22]. Here, we take advantage of ^1^H-NMR to characterize the metabolome of urine and kidney lysates after mechanical I/R in mice at increasing time point *post* reperfusion. In addition to the identification of specific metabolites affected by renal I/R, we plot the correlation between the metabolomic signature and levels of serum creatinine and urea regarded as a conventional markers of I/R severity.

## Materials and Methods

### Acute renal ischemia in mice

This study was carried out in strict accordance with the recommendations in the Guide for the Care and Use of Laboratory Animals of the National Institutes of Health. The protocol was approved by the Committee on the Ethics of Animal Experiments of the University of Liège School of Medicine (protocol number #1335). All surgery was performed under anesthesia, and all efforts were made to minimize suffering. Ten-week-old male C57BL/6 mice weighing ~20g were anesthetized with pentobarbital (60 mg/kg, Ceva^®^) by i.p. injection and, using aseptic techniques, subjected to a laparotomy with bilateral renal pedicle clamping (“ischemia”) for 30 min. Ischemia was confirmed by color change observed in kidneys following clamping. Supportive fluids were given throughout the operative period, and hypothermia was prevented by use of an isothermal heating pad and warming lights.

### Urine and blood collection

Following surgery, animals were kept in light- and temperature-controlled conditions for maximum 48 hours, with a twice-daily clinical evaluation of scar and general health status. Mice were placed in metabolic cages for maximum 24 hours with *ad libitum* access to food and drinking water. Urine was collected on 2%-Na^+^ azide solution (Sigma^®^) with one drop of mineral oil (Sigma^®^) to prevent bacterial proliferation and evaporation, respectively. Blood was obtained by *vena cava* puncture at the time of sacrifice, kept at room temperature for 2h, and centrifuged (10.000r/min. for 10 min) to collect supernatants (sera) for storage at -20°C. Serum levels of urea and creatinine were measured on a COBAS 6000 C501 device (Roche-Hitachi^®^). Kidneys were snap-frozen and stored at -80°C. Urine, serum and kidney metabolome were analyzed using ^1^H-NMR (*see infra*).

### Study groups

Renal tissue, urine and serum were examined in 3 different groups: 6h, 24h and 48h *post*-reperfusion. 27 (13 sham and 14 I/R), 21 (10 sham and 11 I/R) and 32 (13 sham and 19 I/R) kidney samples were collected at 6, 24 and 48h post-reperfusion, respectively. Concerning urine samples, 24 (11 sham and 13 I/R) at 6h, 31 (17 sham and 14 I/R) at 24h and 31 (15 sham and 16 I/R) at 48h *post*-reperfusion were collected. Finally the number of sera samples are of 27 (14 sham and 13 I/R) at 6h, 20 (10 sham and 10 I/R) at 24h and 32 (13 sham and 19 I/R) at 48h *post*-reperfusion.

### ^1^H-NMR metabolomics

All samples were recorded at 298 K on a Bruker Avance spectrometer operating at 500.13 MHz for the proton signal acquisition. The instrument was equipped with a 5 mm TCI cryoprobe with a Z-gradient. Maleic acid was used as internal standard for quantification and trimethylsilyl-3-propionic acid-*d*4 (TMSP) for the zero calibration. Upper half left of each kidney (±50mg) was suspended in 700 μl of deuterated phosphate buffer (DPB, pH 7.4), placed in a 2ml centrifugation vial in an ice bath and then subjected to sonication with the vibrating probe (Vibra-Cell, Sonics and Materials Inc., Newtown, USA) for periods of 2 x 30s. The mixture was then centrifuged (13.300 r/min, 4°C for 10 min) to eliminate membranes and cell residues, and 600 μL of the supernatant was supplemented with 100 μl of a 5 mM solution of maleic acid and 10 μl of a 10 mg/ml TMSP D2O solution. Serum (100 μl) and urine samples (150 μl) were supplemented with 400 (serum) or 450 (urine) μl of deuterated phosphate buffer (DPB, pH 7.4), 100 μl of a 5 mM solution of maleic acid and 10 μl of a 10 mg/ml TMSP solution. ^1^H-NMR spectra were acquired using a 1D NOESY sequence with presaturation for urine samples and CPMG relaxation-editing sequence with presaturation for serum samples and kidney lysates. The Noesypresat experiment used a RD-90°-T_1_-90°-T_m_-90°-acquire sequence with a relaxation delay of 4 s, a mixing time (T_m_) of 10 ms and a fixed T_1_ delay of 4 μs. Water suppression pulse was placed during the relaxation delay (RD). The number of transient is 128 (64K data points) for urine and kidney lysates, 32 for serum samples and a number of 4 dummy scans is chosen. Acquisition time is fixed to 3.2769001 s. The CPMG experiment used a RD-90-(t-180-t)n-sequence with a relaxation delay (RD) of 2s, a spin echo delay (t) of 400 ms and the number of loops (n) equal to 80. The water suppression pulse was placed during the relaxation delay (RD). The number of transients was typically 32. The acquisition time was set to 3.982555 s and a quantity of four dummy scans was chosen. The data were processed with the Bruker Topspin 3.1 software with a standard parameter set. Phase and baseline corrections were performed manually over the entire range of the spectra and the δ scale was calibrated to 0 ppm using the internal standard TMSP.

### Multivariate analysis

For statistical analysis, optimized ^1^H-NMR spectra were automatically baseline-corrected and reduced to ASCII files using AMIX software (version 3.9.14; Bruker). The spectral intensities were normalized to total intensities and reduced to integrated regions of equal width (0.04 ppm) corresponding to the 0.5–10.00 ppm region. Because of the residual signals of water and maleic acid, regions between 4.7 and 5 ppm (water signal) and 5.6–6.2 ppm (maleic acid signal) were removed before analysis. The reduced and normalized NMR spectral data were imported into SIMCA (version 13.0.3, Umetrics AB, Umea Sweden). Pareto scaling was applied to bucket tables and discriminant analysis (DA) such as PCA (Principal Component Analysis), PLS-DA (Partial Least Squares Discriminant Analysis), OPLS-DA (orthogonal partial least squares discriminant analysis) and PLS (Partial Least Square) regression were performed. SIMCA was used to generate all PCA, PLS, PLS-DA, and OPLS-DA models and plots. PCA was only used to detect possible outliers and determine intrinsic clusters within the data set, while PLS-DA maximized the separation and OPLS-DA facilitated the graphic visualization of differences and similarities between groups. The quality of OPLS-DA models was determined by the goodness of fit (R²) and the predictability was calculated on the basis of the fraction correctly predicted in one-seventh cross-validation (Q²). By using the web-based analysis tool, Metaboanalyst (www.metaboanalyst.ca), receiver operating characteristic (ROC) curves were drawn to assess the robustness of the models. ROC analyses were based on PLS-DA models as classification methods with 3 latent variables. Model sensitivity and specificity were calculated from the ROC confusion matrix (generated on the basis of the average of predicted class probabilities of each sample across 100 cross-validations). ROC curves were generated by Monte-Carlo cross validation (MCCV) using balanced sub-sampling. In each MCCV, two thirds (2/3) of the samples were used to evaluate the feature importance. The top 100 important features were then used to build classification models which were validated on the residual 1/3 sample. The procedure was repeated multiple times to calculate performance and confidence intervals of each model.

### Metabolite identification

From PLS-DA and OPLS-DA loading plots, metabolites with higher loadings were identified. Signals with values of Variable Importance in Projection (VIP) higher than 1 were considered as significant, and further validated using *t*-test with Metaboanalyst. Metabolite identification was next performed using the open-access database NMR suite 8.1 (Chenomx inc., Edmonton, Canada), the free web-based tool HMDB (http://www.hmdb.ca) and tables. Each metabolite identified was finally confirmed by performing peak correlation plots from 2D-NMR spectra (COSY and HSQC).

### Pathway analysis

The detailed analysis of the metabolic pathways were performed by Metaboanalyst (www.metaboanalyst.ca) using the Metabolic Set Enrichment Analysis (MSEA) with an Over Representation Analysis (ORA) algorithm. MSEA is a metabolomic version of the Gene Set Enrichment Analysis (GSEA) and aims at identifying patterns of metabolites. It contrasts with the conventional approach in which each metabolite is individually evaluated, thereby improving the identification of subtle and coordinated changes in related compounds [23].

## Results

### Thirty minutes of bilateral renal I/R induce recoverable AKI

**Fig 1:**
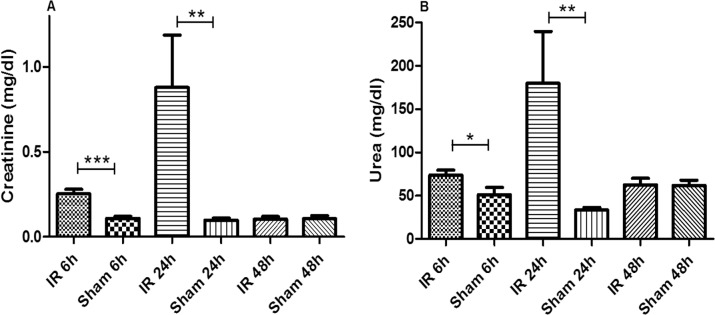
**Serum levels of creatinine (A) and blood urea (B) in mice following renal ischemia/reperfusion or sham surgery**. Unpaired Student t-test between groups of renal ischemia/reperfusion (I/R) and sham surgery showed statistically significant differences (*, *p<0*.05; **, *p<0*.01; ***, *p<0*.*001*) for both serum creatinine and urea levels at 6h and 24h *post* reperfusion.

Ten-week-old male C57BL/6 mice were subjected to 30 minutes of bilateral renal ischemia followed by 6h, 24h, and 48h of reperfusion. The renal function was monitored by serum levels of creatinine and urea (Fig 1). In comparison to sham-operated animals (creatinine, 0.11 ± 0.03; urea, 41.9 ± 6.4 mg/dl), I/R-exposed mice showed a significant increase of both AKI parameters, as early as 6h *post* reperfusion (creatinine, 0.26 ± 0.09; urea, 75.7 ± 22.4 mg/dl). The levels of creatinine and urea peaked at 24h *post* reperfusion (creatinine, 0.88 ± 0.69; urea, 179.6 ± 60.1 mg/dl). At 48h *post* I/R, levels of both AKI parameters significantly recovered (creatinine, 0.11 ± 0.04; urea, 62.5 ± 22.7 mg/dl). Such an experimental model of transient I/R-induced AKI allows determining early metabolomic changes during initial and peak phases of injury.

### Thirty minutes of bilateral renal I/R induce early and significant changes in urine and kidney metabolomes, but not in serum profile

**Fig 2:**
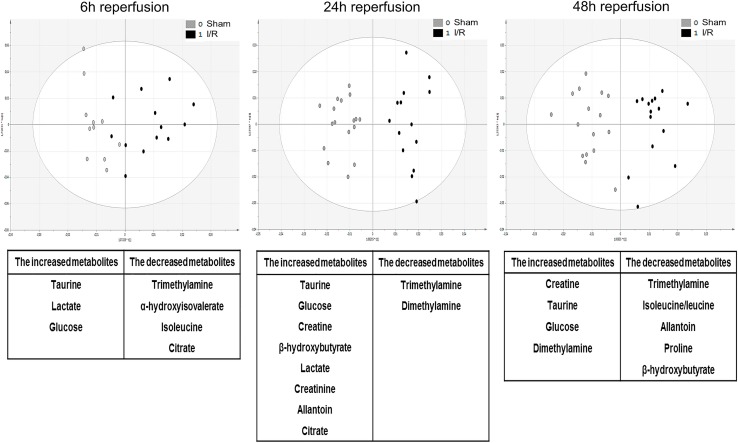
Score plots from OPLS-DA applied to ^1^H-NMR spectra of mouse urine samples following renal ischemia/reperfusion or sham surgery. The upper panels represent the score plots of OPLS-DA from ^1^H-NMR metabolomic analysis using mouse urine samples collected after 6-hour (left), 24-hour (middle) and 48-hour (right) reperfusion following renal ischemia (black dots) or sham surgery (grey dots). The lower tables correspondingly list the metabolites whose urinary abundance is significantly increased or decreased after renal ischemia/reperfusion (I/R) in comparison to sham surgery.

**Fig 3:**
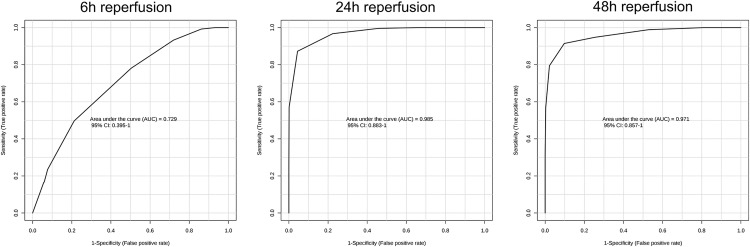
Receiver operating characteristic (ROC) curves of ^1^H-NMR metabolomics of mouse urine samples following renal ischemia/reperfusion or sham surgery. Multivariate ROC curves were drawn using ^1^H-NMR metabolomic spectral data from mouse urine samples collected after 6-hour (left), 24-hour (middle) and 48-hour (right) reperfusion following renal ischemia.

**Fig 4:**
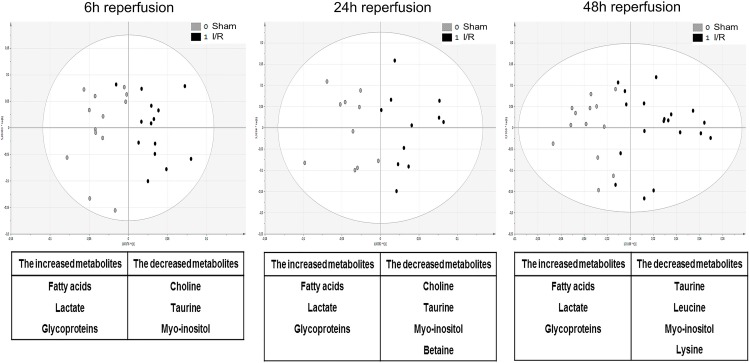
Score plot from OPLS-DA applied to ^1^H-NMR spectra of mouse kidney lysates following renal ischemia/reperfusion or sham surgery. The upper panels represent the score plots of OPLS-DA from ^1^H-NMR metabolomic analysis using mouse kidney lysates collected after 6-hour (left), 24-hour (middle) and 48-hour (right) reperfusion following renal ischemia (black dots) or sham surgery (grey dots). The lower tables correspondingly list the metabolites whose abundance in renal parenchyma is significantly increased or decreased after renal ischemia/reperfusion (I/R) in comparison to sham surgery.

**Fig 5:**
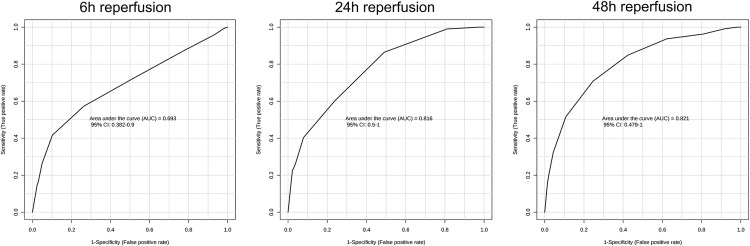
Receiver operating characteristic (ROC) curves of ^1^H-NMR metabolomics of mouse kidney samples following renal ischemia/reperfusion or sham surgery. Multivariate ROC curves were drawn using ^1^H-NMR metabolomic spectral data from mouse kidney samples collected after 6-hour (left), 24-hour (middle) and 48-hour (right) reperfusion following renal ischemia.

Urine samples were initially collected before the I/R operation, but were not used as references since score plots from OPLS-DA applied to ^1^H-NMR spectra highlighted a significant impact of sham surgery *per se* on urine metabolome (S1 Fig). Therefore, urine samples were collected in both sham-operated and I/R-exposed mice at 6h, 24h and 48h *post* surgery, and processed following ^1^H-NMR metabolomic procedure (S1–S3 Datasets). Representative spectra of each time-point *post* reperfusion are shown in S2 Fig. Statistical investigations using OPLS-DA represented as score plots (Fig 2; upper panels) highlighted a significant discrimination of sham-operated *versus* I/R-exposed groups as early as 6h *post* surgery (*R*^*2*^: *0*.*651*, *Q*^*2*^: *0*.*153*), which was sustained at 24h (*R*^*2*^: *0*.*892*,*Q*^*2*^: *0*.*740*) and 48h (*R*^*2*^: *0*.*821*,*Q*^*2*^: *0*.*546*) *post* I/R. Detailed analysis of the associated loading plots allowed the identification of metabolites of increased or decreased abundance in I/R group in comparison to sham-operated mice (Fig 2; lower panels). Hence, urine levels of taurine, lactate and glucose were steadily increased after I/R, whereas urine levels of trimethylamine were significantly reduced. Model validity was assessed by ROC analysis (based on PLS-DA model), as described above. Sensitivity and specificity of the model respectively reached 60% and 76.9% at 6h *post*-IR, 93.7% and 100% at 24h, 94.4% and 94.1% at 48h. Area under the ROC curve (AUC) at 24h and 48h post-surgery were 0.98 and 0.97 respectively, suggesting a high predictive accuracy. Conversely, the AUC at 6h post-surgery was 0.73 (Fig 3). MSEA analysis led to the identification of several pathways significantly affected by renal I/R. The most relevant cascades were gluconeogenesis and taurine / hypotaurine metabolism at 6 and 24h reperfusion. Protein biosynthesis, glycolysis and galactose and arginine metabolisms appeared essential at 48h reperfusion. Next, kidney samples were similarly collected in both sham-operated and I/R-exposed mice at 6h, 24h and 48h *post* surgery, lysed, and processed by ^1^H-NMR metabolomics (S4–S6 Datasets). Representative spectra of each time-point *post* reperfusion are shown in S3 Fig. OPLS-DA kidney analysis found a significant discrimination between sham-operated and I/R-exposed animals at 6h (*R*^*2*^: *0*.*692*, *Q*^*2*^: *0*.*388*), 24h (*R*^*2*^: *0*.*705*, *Q*^*2*^: *0*.*441*) and 48h (*R*^*2*^: *0*.*643*, *Q*_*2*_:*0*.*416*) *post* surgery (Fig 4; upper panels). The identification of metabolites, whose increased abundance reached significance in loading plots included fatty acids (and modified lipoproteins), lactate and N-acetyl groups of glycoproteins. Conversely, levels of taurine and myo-inositol were decreased in kidneys from I/R-exposed mice in comparison to sham-operated animals (Fig 4; lower panels). Sensitivity and specificity of the model were respectively 73.3% and 56.2% at 6h *post*-IR, 72.7% and 63.6% at 24h, 68.4% and 78.5% at 48h. The AUC of the ROC curves (based on PLS-DA models) at 6h, 24h and 48h post-surgery reached 0.69, 0.82 and 0.82, respectively (Fig 5). MSEA analysis of metabolites at 6h and 24h reperfusion revealed that taurine / hypotaurine and betaine metabolisms were significantly affected by renal I/R. At 48h *post* reperfusion, I/R-associated cascades were protein biosynthesis, biotin and taurine / hypotaurine metabolisms. Finally, ^1^H-NMR metabolomic analysis of sera collected at 6h, 24h and 48h *post* surgery could not discriminate sham-operated from I/R-exposed animals (*data not shown*). Representative spectra of each time-point *post* reperfusion are shown in S4 Fig. Of interesting note, the signals corresponding to urea and creatinine in serum samples are barely detectable in rodents, in strong contrast to man (S5 Fig). This may be linked to species variability and/or ^1^H-NMR sensitivity/specificity. Also, our experimental handling of serum samples may not be the most adapted for ^1^H-NMR.

### Urine metabolome significantly correlates with serum levels of creatinine and urea

Transversal correlation studies were performed between urine metabolome and serum levels of creatinine or urea at increasing time points following renal I/R. These correlations were performed by PLS regression using all NMR spectral regions included in the original OPLS-DA. As a reminder, regions between 4.7 and 5 ppm and 5.6–6.2 ppm were removed because of the residual signals of water and maleic acid, respectively. At 6h *post* surgery, a significant positive correlation was found between urine metabolome and serum levels of urea (*r²*, *0*.*69; Q*_*2*_, *0*.*352*) and creatinine (*r²*, *0*.*87; Q*_*2*_, *0*.*117*). Such a positive correlation between urine metabolome and AKI parameters was further observed at 24h (urea (*r²*, *0*.*93; Q*_*2*_, *0*.*693*) and creatinine (*r²*, *0*.*95; Q*_*2*_, *0*.*817*)) and at 48h (urea (*r²*, *0*.*94; Q*_*2*_, *0*.*154*); creatinine (*r²*, *0*.*98; Q*_*2*_, *772*)) *post* surgery.

## Discussion

Renal I/R is the leading cause of AKI, which represents a frequent morbid condition, particularly in operative settings like cardio-thoracic surgery and solid organ transplantation [2, 24]. Advances in deciphering the pathophysiology of renal I/R injury are urgently required to fill the gap of preventive and curative approaches in AKI [25, 26]. Metabolomics presents an innovative method in (patho)physiology, which empowers us to comprehensively and systematically study and characterize metabolite changes in biological systems in response to perturbation [8, 27]. Metabolomics is by nature hypothesis-generating. Indeed, adopting such a systems biology approach will (i) facilitate our understanding of mechanistic pathways that play a role in I/R, (ii) allow for biomarker discovery and earlier AKI diagnosis, and (iii) assist the monitoring of efficacy and toxicity of current and future therapeutic approaches to prevent and eventually cure AKI [14, 15]. ^1^H-NMR spectroscopy is regarded as quantitative given that the intensity of a signal is directly related to the amount of resonant nuclei. However, due to the abundance of water in our samples, water signal suppression had to be performed, which may affect the baseline, as well as signal intensities. Additionally, signal overlapping hampers the integration step. Therefore, comparative analysis was rather chosen here.

Our murine model of bilateral renal I/R produced early, robust and dynamic changes of metabolites in urine and kidney parenchyma over time. By contrast, no significant change was observed in serum samples following renal I/R using ^1^H-NMR metabolomics. Of note, serum was collected and worked-up following the classical protocol used for medical chemistry, which might not be appropriate for ^1^H-NMR approach. In early phase of I/R-induced AKI, i.e. 6h *post* surgery, both urine and kidney metabolome showed evidence of altered energy metabolism affecting glycolysis, tricarboxylic acid cycle and lipid metabolism. The accumulation of lactate, end-product of anaerobic glycolysis, was particularly observed in both urine and kidney lysates. Anoxia, ischemia, and infarction produce rapid loss of high-energy phosphates and accumulation of hydrolysis products, like lactate, β-hydroxybutyrate and citrate [28]. Beside the metabolic cascades, renal I/R appears to significantly impact osmoregulation, as reflected by the altered levels of osmolytes, like taurine, betaine and trimethylamine. Organic osmolytes are small solutes used by cells of numerous water-stressed organisms and tissues to maintain cell volume [29]. They include amino acids and derivatives, polyols and sugars, methylamines, methylsulfonium compounds and urea. Here, taurine appeared to be significantly lost into the urine following I/R, with a decreased abundance in renal parenchyma. In a rat model of renal I/R, prior i.v. administration of taurine significantly reduces injury, as reflected by final serum creatinine levels much lower than in control rats [30]. No protection in terms of ATP content was found. Likewise, the addition of taurine to organ preservation solution was able to reduce tissue alterations during hypoxia and reoxygenation and permitted recovery of energy metabolism in LLC-PK1 cells [31]. In addition to its role in osmoregulation, the most important function for taurine in oxidant I/R likely involves the local and systemic scavenging of reactive oxygen species [32, 33]. In contrast to taurine’s metabolomic profile, the urinary abundance of another organic osmolyte, i.e. trimethylamine (TMA), appeared reduced following renal I/R. TMA is a volatile tertiary aliphatic amine that is derived from the diet either directly from the consumption of foods containing TMA, or by the intake of food containing precursors to TMA such as trimethylamine-N-oxide (TMAO), choline and L-carnitine[34]. In the mammalian kidney, TMA may help enhance protein folding and ligand binding in order to counteract perturbations by urea, inorganic ions, and hydrostatic pressure [29, 34]. In murine and porcine models of severe I/R induced by kidney transplantation, elevations of TMA levels in blood and urine detected by ^1^H-NMR spectroscopy have been identified as reliable markers of renal medullary injury [22, 35]. Furthermore, renal graft dysfunction is associated with damage to the renal medulla as determined by TMA release in urine and plasma [35]. Finally, renal I/R induced a significant loss of betaine, a key choline-derivated osmolyte of kidney medulla [36]. Previous studies similarly pointed towards an impact of I/R-induced AKI on betain homeostasis [20, 37]. Altogether, these observations suggest that renal I/R significantly modifies osmoregulation and its associated biochemical pathways. The exact underlying mechanisms taking place in renal parenchyma and tubules require further investigation.

Our present NMR-based metabolomic approach does not technically allow us establish the exact nature of glycoproteins and lipids impacted by renal I/R. Still, in kidney samples, spectral data disclosed significant variations in signals related to methyl- and methylene groups of fatty acids (but not in phospholipids and sphingolipids), as well as in acetyl groups linked to glycoproteins. These metabolomics changes are not observed in blood and urine samples. Major lipids play key roles in membrane bilayer structure, signaling pathways and energy storage, and provide functional support to membrane proteins [38]. I/R-associated AKI induces cellular membrane instability, leading to lipid dysfunction and accumulation in the renal parenchyma. There, lipids may be either protective or toxic depending on their species and the time-course of the injury [39]. Further “lipidomic” studies may help better understand such impact of renal I/R injury on lipid compositions and functions [40].

A major debate is ongoing regarding the need for normalization of urinary biomarker concentration to urinary creatinine (UCr) [41, 42]. Normalization to UCr appears to be valid for the evaluation of chronic kidney disease, but may be inappropriate in case of acute conditions. Therefore, in our present study, normalization of urine metabolite concentrations was done upon the global metabolome and not to UCr. The correlation of the urine metabolomic profile measured by ^1^H-NMR spectroscopy with classical biomarkers of I/R-induced AKI, i.e. SCr and urea, was determined by multivariate linear regression analysis using PLS approach. The AUC of urine metabolome at 24h and 48h *post* surgery reached 0.98 and 0.97 respectively, suggesting a high predictive accuracy. After validation in man, one may expect that urine metabolome analysis may help noninvasively follow kidney function, and eventually detect renal dysfunction at early stages.

## Conclusion

Our study demonstrates that ^1^H-NMR metabolomics is able to identify early and sustained metabolic changes in urine and kidney composition, but not in serum, caused by renal I/R. The most implicated pathways at 6 and 24h *post* reperfusion include gluconeogenesis, taurine / hypotaurine metabolism, whereas protein biosynthesis, glycolysis, and galactose and arginine metabolisms are key at 48h *post* reperfusion. Such innovative approach may open new research avenues in the understanding, diagnosis and prevention of I/R-associated AKI.

## Supporting Information

S1 Dataset NMR-based metabolomics of urine samples collected at 6h post reperfusion. (TXT)

S2 Dataset NMR-based metabolomics of urine samples collected at 24h post reperfusion. (TXT)

S3 Dataset NMR-based metabolomics of urine samples collected at 48h post reperfusion. (TXT)

S4 Dataset NMR-based metabolomics of kidney samples collected at 6h post reperfusion. (TXT)

S5 Dataset NMR-based metabolomics of kidney samples collected at 24h post reperfusion. (TXT)

S6 Dataset NMR-based metabolomics of kidney samples collected at 48h post reperfusion. (TXT)

S1 Fig Score plots from OPLS-DA applied to ^1^H-NMR spectra of mouse urine samples collected before (light grey dots) *versus* 24h (panel A) or 48h (panel B) after renal ischemia/reperfusion (black dots) or sham surgery (dark grey dots).
(TIF)

S2 Fig **^1^H-NMR spectra of mouse urine at 6h (A), 24h (B) and 48h (C) *post* reperfusion.** Relevant metabolites are identified by letters, which correspond to a, isoleucine/leucine; b, lactate; c, proline; d, citrate; e, dimethylamine; f, trimethylamine; g, creatine; h, taurine; i, creatinine; j, glucose; k, allantoin.
(TIF)

S3 Fig **^1^H-NMR spectra of mouse kidney at 6h (A), 24h (B) and 48h (C) *post* reperfusion.** Relevant metabolites are identified by letters, which corresponds to a, methyl and methylene protons of fatty acid chains; b, lactate; c, N-acetyl groups of glycoproteins; d, choline; e, myoinositol; f, taurine.
(TIF)

S4 Fig ^1^H-NMR spectra of mouse serum at 6h (A), 24h (B) and 48h (C) *post* reperfusion.
(TIF)

S5 Fig **^1^H-NMR spectra of human (A), rat (B) and mouse (C) serum.** Spectral regions corresponding to creatinine (*) and urea (**) are indicated by grey zones.
(TIF)
