# Supplementary figures and images for: Nuclear Magnetic Resonance Metabolomic Profiling of Mouse Kidney, Urine and Serum Following Renal Ischemia/Reperfusion Injury
Source: PLoS One. 2016 Sep 22;11(9):e0163021. doi: 10.1371/journal.pone.0163021 (PMC5033333; doi:10.1371/journal.pone.0163021)

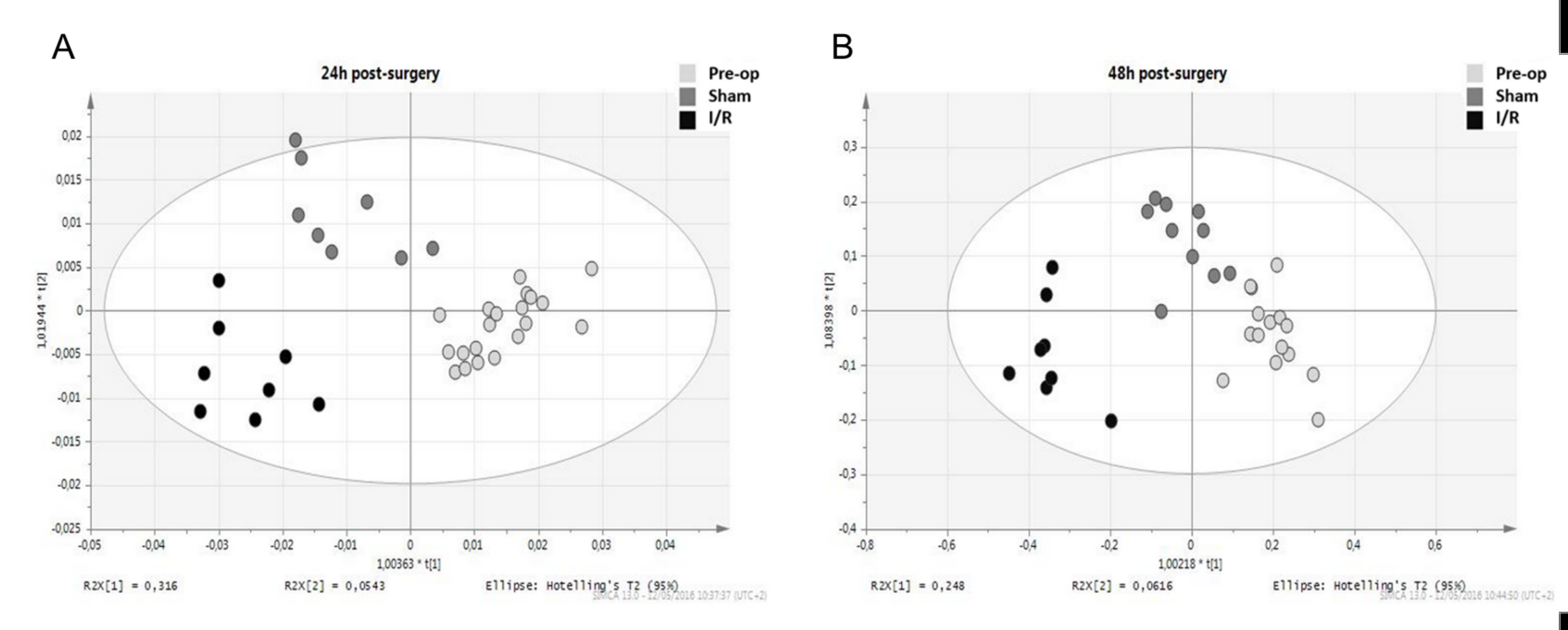

Supplement: S1 Fig — Score plots from OPLS-DA applied to 1H-NMR spectra of mouse urine samples collected before (light grey dots) versus 24h (panel A) or 48h (panel B) after renal ischemia/reperfusion (black dots) or sham surgery (dark grey dots). (TIF) [file pone.0163021.s007.tif]

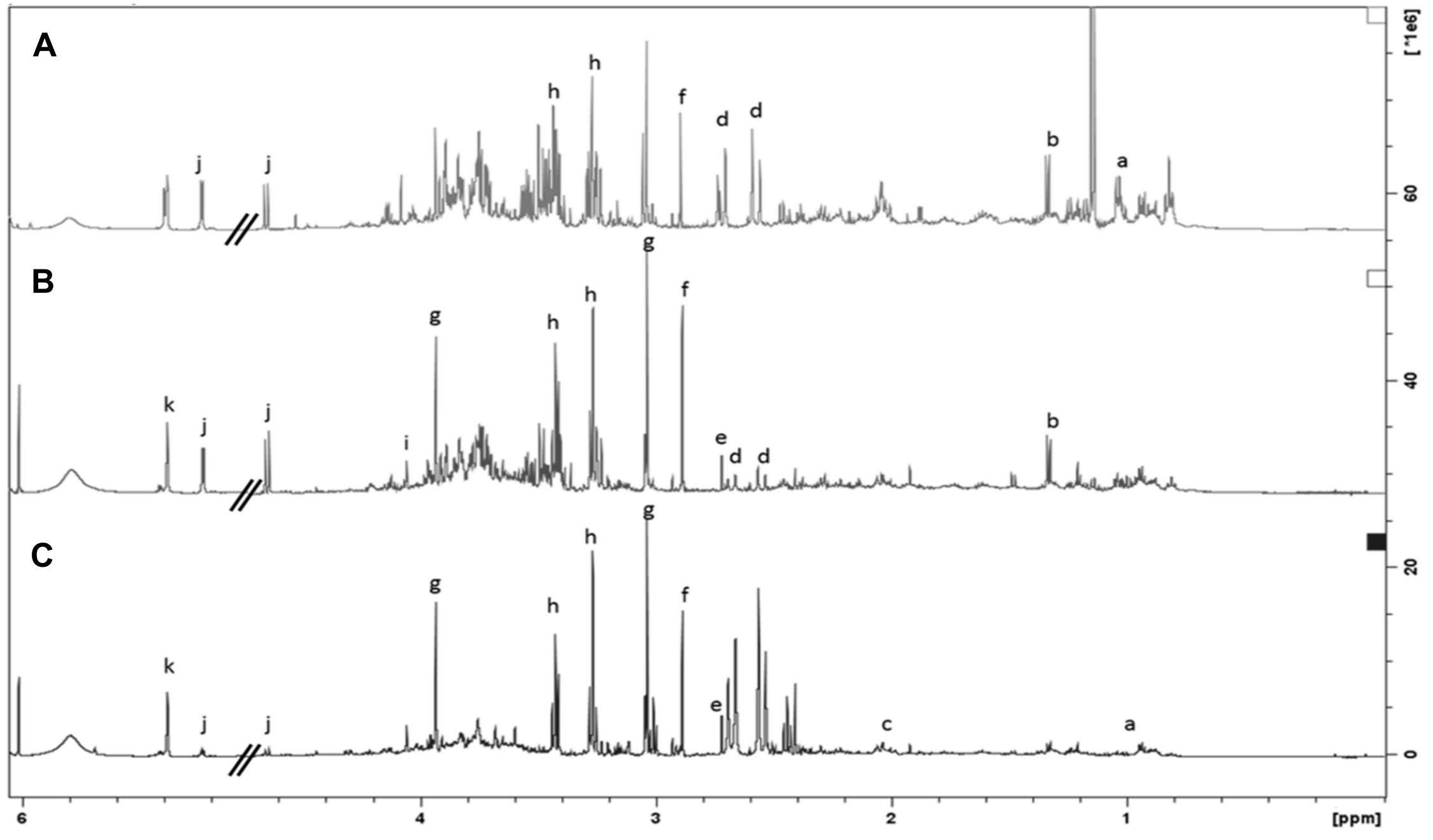

Supplement: S2 Fig — 1H-NMR spectra of mouse urine at 6h (A), 24h (B) and 48h (C) post reperfusion. Relevant metabolites are identified by letters, which correspond to a, isoleucine/leucine; b, lactate; c, proline; d, citrate; e, dimethylamine; f, trimethylamine; g, creatine; h, taurine; i, creatinine; j, glucose; k, allantoin. (TIF) [file pone.0163021.s008.tif]

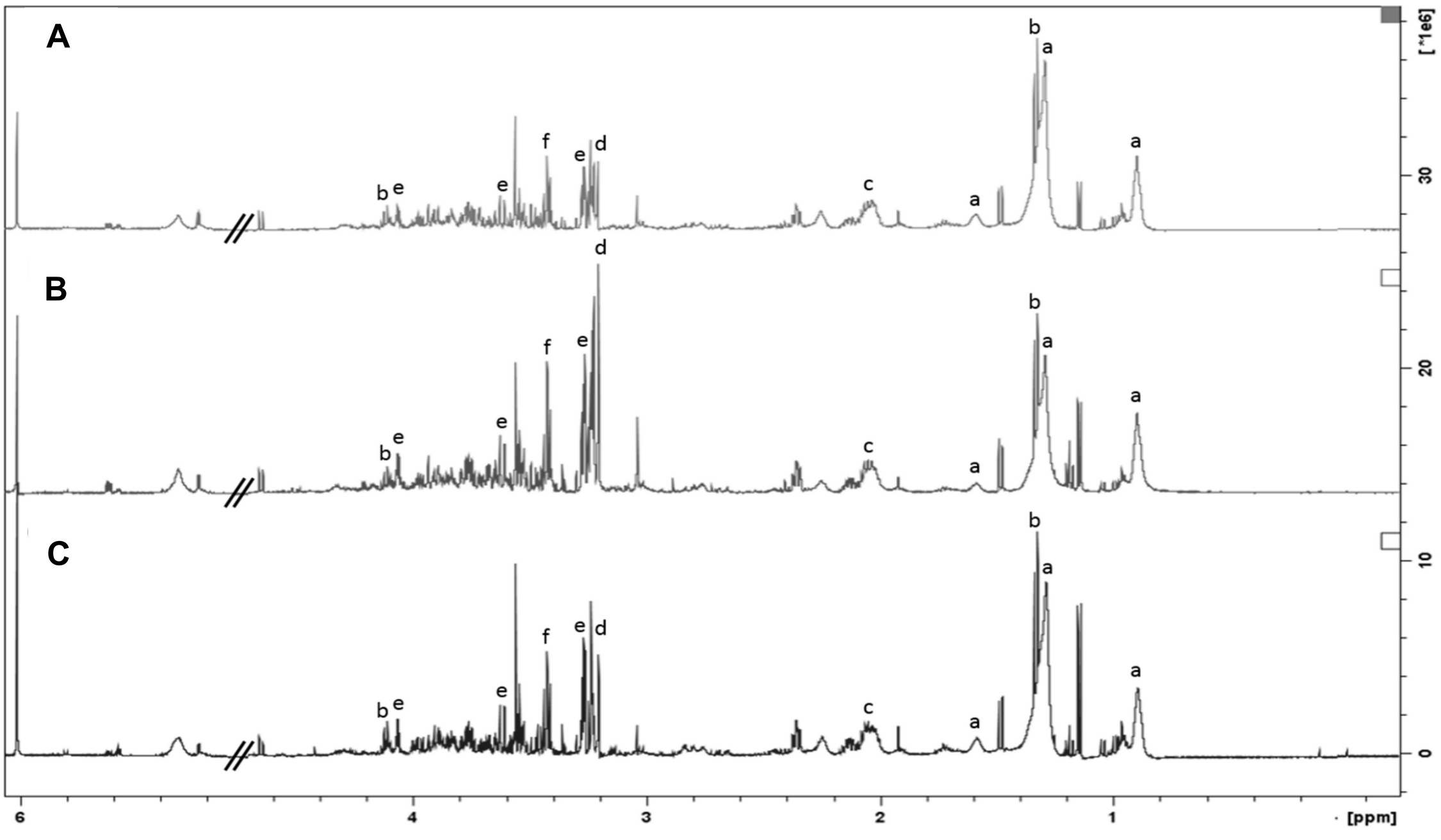

Supplement: S3 Fig — 1H-NMR spectra of mouse kidney at 6h (A), 24h (B) and 48h (C) post reperfusion. Relevant metabolites are identified by letters, which corresponds to a, methyl and methylene protons of fatty acid chains; b, lactate; c, N-acetyl groups of glycoproteins; d, choline; e, myoinositol; f, taurine. (TIF) [file pone.0163021.s009.tif]

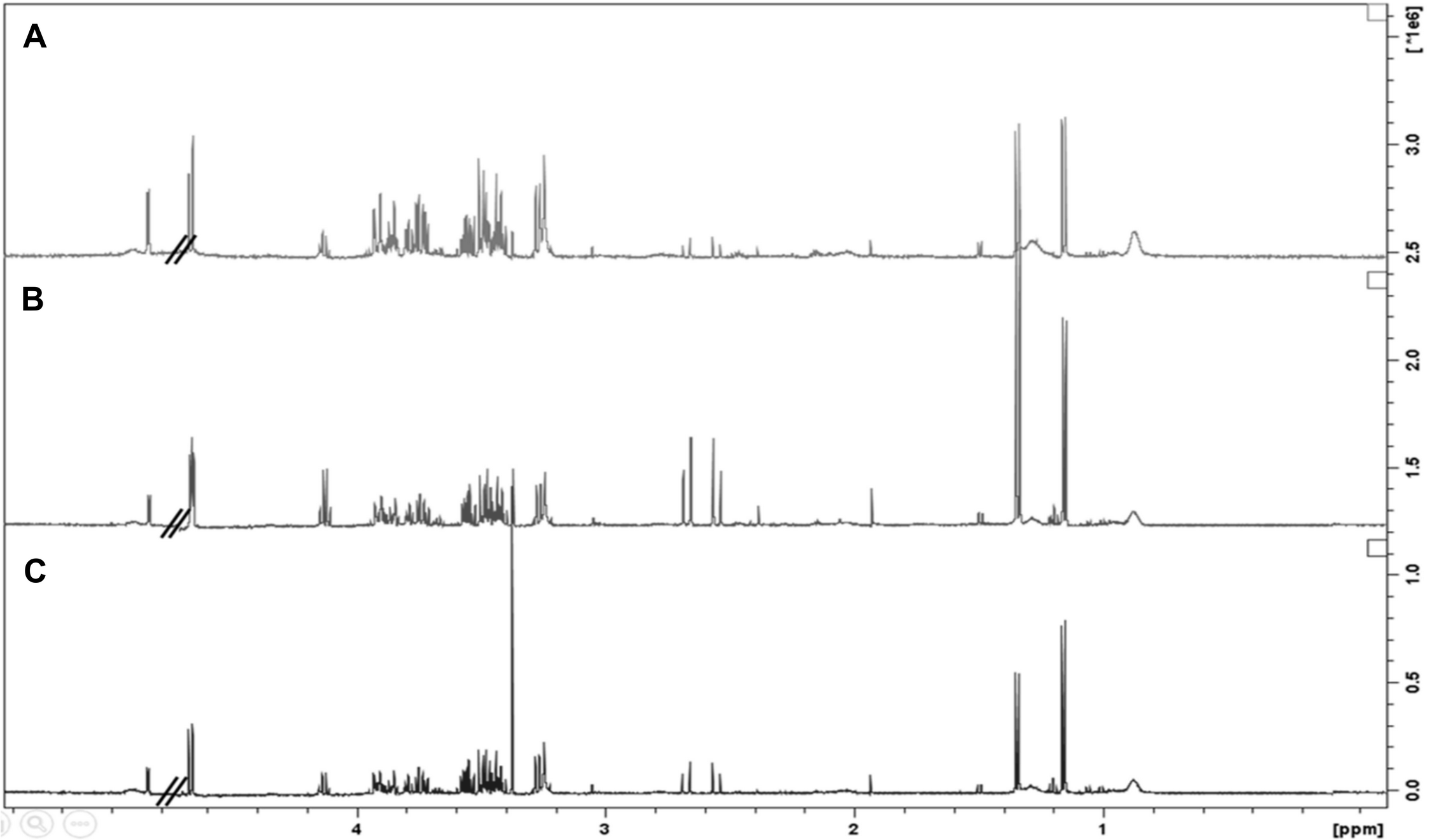

Supplement: S4 Fig — 1H-NMR spectra of mouse serum at 6h (A), 24h (B) and 48h (C) post reperfusion. (TIF) [file pone.0163021.s010.tif]

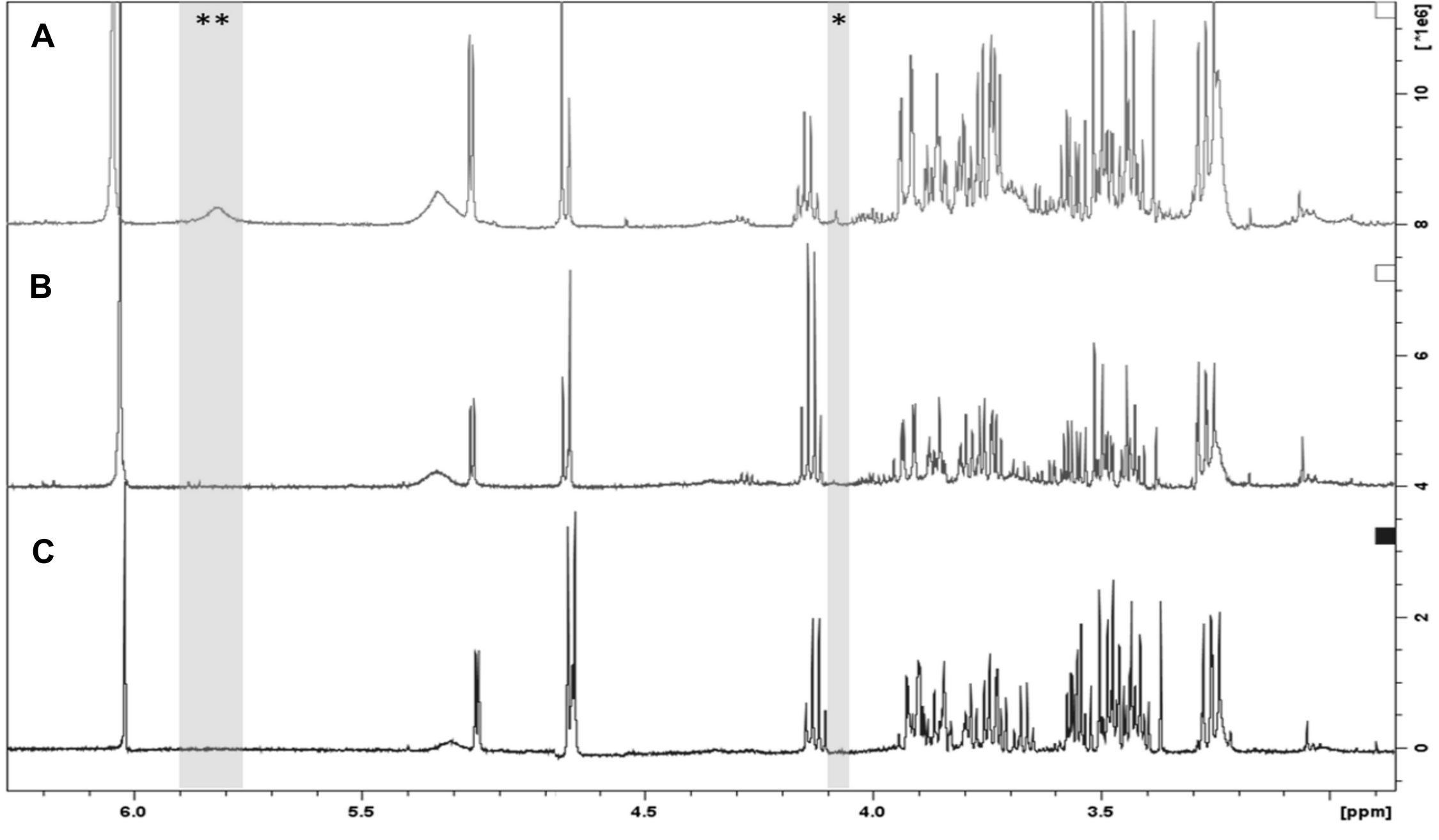

Supplement: S5 Fig — 1H-NMR spectra of human (A), rat (B) and mouse (C) serum. Spectral regions corresponding to creatinine (*) and urea (**) are indicated by grey zones. (TIF) [file pone.0163021.s011.tif]
